# PREVALENCE OF *HELICOBACTER PYLORI* INFECTION AMONG GASTROENTEROLOGISTS AND GASTROENDOSCOPISTS IN BRAZIL

**DOI:** 10.1590/S0004-2803.24612025-019

**Published:** 2025-09-05

**Authors:** Luiz Gonzaga Vaz COELHO, Décio CHINZON, Laércio Tenório RIBEIRO, Bruno Squárcio Fernandes SANCHES, Áureo de Almeida DELGADO, Eduardo Garcia VILELA

**Affiliations:** 1 Hospital das Clínicas da Universidade Federal de Minas Gerais/Ebserh, Instituto Alfa de Gastroenterologia; Belo Horizonte, MG, Brasil.; 2 Hospital das Clínicas da Universidade de São Paulo, Departamento de Gastroenterologia, São Paulo, SP, Brasil.; 3 Hospital Universitário Professor Alberto Antunes, Divisão de Endoscopia, Maceió, AL, Brasil.; 4 Faculdade de Medicina da Universidade Federal de Minas Gerais, Programa de Ciência Aplicadas à Saúde do Adulto, Centro de Pós-Graduação, Residência Pós-Doutoral, Belo Horizonte, MG, Brasil.; 5 Faculdade de Medicina da Universidade Federal de Juiz de Fora, Juiz de Fora, MG, Brasil.

**Keywords:** Prevalence, 13C-urea breath test, Helicobacter pylori, gastroenterologists, endoscopic procedures, epidemiology, Brazil, Prevalência, teste respiratório com 13C-ureia, Helicobacter pylori, gastroenterologistas, procedimentos endoscópicos, epidemiologia, Brasil

## Abstract

**Background:**

Most *Helicobacter pylori* (*H. pylori*) infections are acquired in childhood. It remains uncertain whether gastroenterologists involved in endoscopic procedures face an increased occupational risk of *H. pylori*.

**Objective:**

To determine *H. pylori* prevalence among gastroenterologists and gastroendoscopists in Brazil.

**Methods:**

A prospective, observational, non-interventional study was conducted during the 2022 Brazilian Digestive Disease Week meeting. Attendees were invited to undergo a 13C-urea breath test (UBT) to investigate their *H. pylori* status. The attendees completed a questionnaire regarding their demographic data and information about medical specialties and activities (gastroenterology or gastroendoscopy). This study included 286 participants (160 women, 126 men; mean age, 42 years; SD, 13, range 25-83 years) agreed to participate. 13C-urea breath test: Before the study, all participants abstained from proton pump inhibitors (PPIs) and H2 blockers for 1 week, and antibiotics for four weeks. The test was performed after at least one-hour of fasting using the BreathID HP Lab System^®^ (Exalenz Bioscience, Israel, now Meridian Bioscience, USA), with a delta over baseline (DOB) ≥5‰ indicated *H. pylori* infection.

**Results:**

Among the 286 study participants, 218 tested negative and 68 tested positive with an overall prevalence of 23.8%. If we excluded all 67 participants who reported prior treatment for HP infection (54 HP-ve and 13 HP+ve) from the analysis of our sample, our sample of 219 participants presented a current prevalence of 25.1% (55 HP+ve and 164 HP-ve). The HP prevalence among participants who did or did not perform endoscopic procedures in their daily activities was 28.4% and 23.2%, respectively, with no statistically significant difference (*P*=0.39).

**Conclusion:**

The prevalence of *H. pylori* infection among Brazilian gastroenterologists is moderate, with one in four professionals still infected. *H. pylori* infection prevalence increases with age and is higher among overweight and obese individuals. Performing endoscopic procedures does not appear to increase the risk of infections among gastroenterologists in Brazil.

## HIGHLIGHTS

• It is uncertain whether H. pylori infection constitutes an occupational risk for gastroenterologists performing endoscopic procedures.

• Breath test with carbon-13 labeled urea is a highly accurate method for non-invasive diagnosis of the presence of active H. pylori infection.

• Gastroenterologists and gastroendoscopists present at a large Brazilian event were included in the study.

• Performing endoscopic procedures does not appear to increase the risk of acquiring H. pylori infection among gastroenterologists in Brazil.

## RESUMO

**Introdução:**

A maioria das infecções por *Helicobacter pylori* (*H. pylori*) é adquirida na infância. Permanece incerto se os gastroenterologistas envolvidos em procedimentos endoscópicos enfrentam um risco ocupacional aumentado de infecção por *H. pylori*.

**Objetivo:**

Determinar a prevalência de *H. pylori* entre gastroenterologistas e gastroendoscopistas no Brasil.

**Métodos:**

Um estudo prospectivo, observacional e não intervencionista foi realizado durante a reunião da Semana Brasileira do Aparelho Digestivo de 2022. Os participantes foram convidados a se submeter a um teste respiratório com ureia-marcada com 13C para investigar seu status de *H. pylori*. Os participantes preencheram um questionário com dados demográficos e informações sobre especialidades e atividades médicas (gastroenterologia ou gastroendoscopia). Este estudo incluiu 286 participantes (160 mulheres, 126 homens; idade média, 42 anos; DP, 13, faixa etária de 25 a 83 anos) que concordaram em participar. Teste respiratório com 13C-ureia: Antes do estudo, todos os participantes se abstiveram do uso de inibidores da bomba de prótons e bloqueadores H2 por uma semana e de antibióticos por quatro semanas. O teste foi realizado após pelo menos uma hora de jejum utilizando o BreathID HP Lab System^®^ (Exalenz Bioscience, Israel, agora Meridian Bioscience, EUA), com delta acima da linha de base (DOB) ≥5‰ indicando infecção por *H. pylori*.

**Resultados:**

Entre os 286 participantes do estudo, 218 testaram negativo e 68 testaram positivo, com uma prevalência global de 23,8%. Se excluíssemos todos os 67 participantes que relataram tratamento prévio para infecção por HP (54 HP-ve e 13 HP+ve) da análise de nossa amostra, nossa amostra de 219 participantes apresentou uma prevalência atual de 25,1% (55 HP+ve e 164 HP-ve). A prevalência de *H. pylori* entre os participantes que realizaram ou não procedimentos endoscópicos em suas atividades diárias foi de 28,4% e 23,2%, respectivamente, sem diferença estatisticamente significativa (*P*=0,39).

**Conclusão:**

A prevalência da infecção por *H. pylori* entre gastroenterologistas brasileiros é moderada, com um em cada quatro profissionais ainda infectado. A prevalência da infecção por *H. pylori* aumenta com a idade e é maior entre indivíduos com sobrepeso e obesos. A realização de procedimentos endoscópicos não parece aumentar o risco de infecções entre gastroenterologistas no Brasil.

## INTRODUCTION

*Helicobacter pylori* (*H. pylori*) is recognized as the main etiological agent of chronic gastritis and peptic ulcers, with an equally well-established pathogenic role in gastric adenocarcinoma and MALT (mucosa-associated lymphoid tissue) lymphoma. It may also act as an organic cause of dyspepsia and other extra-gastric diseases^1^ ^,^ ^2^. Identified for the first time in 1983 by Marshall and Warren in Australia^3^, with its first description in Brazil in 1987^4^, it is estimated that it infects approximately 50% of the world’s population today^5^.

Knowledge of *H. pylori* transmission remains incomplete. The likelihood of infection increases with low socioeconomic status and is associated with overcrowded households and poor hygiene. The infection is primarily acquired in childhood and is predominantly transmitted from person to person through the oral route^6^ ^,^ ^7^. *H. pylori* exclusively colonizes the gastric mucosa and has been isolated from feces, gastric juices, vomiting, saliva, and dental plaque^8^ ^-^ ^11^. Several studies have assessed the occupational risk of *H. pylori* infection among health professionals involved in aerodigestive tract procedures, such as dentists, otorhinolaryngologists, pulmonologists, nurses, and gastroenterologists/endoscopists, with controversial results^12^ ^,^ ^13^.

Several diagnostic methods have been developed for detecting *H. pylori* infection, including both invasive and noninvasive tests. Invasive methods, involve upper digestive endoscopy with gastric tissue sampling for histology, culture, urease tests, or molecular tests. Noninvasive methods include serological tests, fecal antigen detection, and carbon-13-labeled urea breath test (UBT). The latter is considered the gold standard for diagnosis, with a sensitivity and specificity greater than 95%^6^ ^,^ ^14^ ^,^ ^15^. Owing to its high accuracy, safety, low cost, and ease of execution, UBT is considered the first option for the diagnosis of active infection and control of treatment or its recurrence. It is also an excellent diagnostic option for small epidemiological studies^6^ ^,^ ^16^. These tests are based on the ability of *H. pylori* to produce high amounts of urease. The test is based on *H. pylori’s* ability to hydrolyze orally absorbed 13C-labeled urea in the gastric environment. The resulting 13CO2 diffuses into the bloodstream and is exhaled, where it can be detected using an infrared spectrophotometer or mass spectrometer^17^. Although validated in Brazil in 1999, its use remains inexplicably limited to a few centers^18^ ^-^ ^22^.

The management of *H. pylori* infections remains a challenge in clinical practice. Treatment recommendations are well established (except in impeding situations) for all diagnosed individuals with infection^2^ ^,^ ^6^ ^,^ ^23^. In countries with high incidence and mortality from gastric cancer, such as Japan, Korea, and Taiwan, treatment programs targeting the entire infected population are being considered as public health preventive measures for gastric cancer^2^ ^,^ ^24^. Additionally, recent studies from China have provided evidence that changing from individual-based to family-based treatment could enhance infection control and related disease prevention^25^ ^,^ ^26^. However, *H. pylori* eradication is becoming increasingly difficult owing to rising antibiotic resistance, and different antimicrobial regimens are now recommended^6^ ^,^ ^16^. The limited availability of non-invasive methods, such as UBT, for diagnosis and post-treatment control of the infection hinders the best approach for patients. Since 1996, the Brazilian Federation of Gastroenterology (FBG) and its Affiliated Association, the Brazilian *Helicobacter pylori* and Microbiota Study Nucleus (NBEHPM), are responsible, for preparing the Brazilian Consensus on *H. pylori* infection^6^ ^,^ ^27^ ^-^ ^29^, among other initiatives aimed at continuously optimizing the management of this condition in the country. This study, conducted by FBG/NBEHPM, aimed to determine the prevalence of *H. pylori* infection among its members and analyze its relationship with different aspects, such as age, sex, BMI, place of birth, and performance of endoscopic procedures.

## METHODS

### Participants

This prospective, observational, non-interventional study was conducted during the XXI Week of the Digestive System (SBAD), an event organized by digestive system-related societies, held from 2022-12-01 to 04, in Florianópolis, the capital of the state of Santa Catarina, Brazil.

For sample size calculation, the formula recommended by Triola^30^ was used for a sample with a known size (N). The population size was defined as 1307, corresponding to the total number of non-defaulting senior members of the FBG (year 2021), and thus distributed in Brazil: Northern region: 109; North Eastern region: 335; Central Western region: 50; Southeastern region: 630; and Southern region: 183. Although no specific studies are available, a 30% *H. pylori* infection has been estimated to be 30%^30^ ^-^ ^32^. Thus, assuming a margin of error of 0.05, the defined sample included 261 gastroenterologists, distributed as follows: Northern region, 22; Northeastern region, 67; Central Western region, 10; Southeastern region, 125; and Southern region, 37. This study was approved by the Research Ethics Committee of the Federal University of Minas Gerais (UFMG) (CAAE: 60031422.7.0000.5149). All the participants signed an informed consent form after clarification.

### Methods

The attendees were invited to undergo a UBT to assess their *H. pylori* status. They were also asked to complete a questionnaire regarding their demographic data and daily gastroenterological practice. To participate in the study, all participants were required to abstain from taking PPI and H2 blockers for 1 week, and antibiotics for 4 weeks, respectively. 

### 13C-urea breath test (UBT)

All study participants underwent the UBT as follows: after at least 1h fasting, exhaled air samples were initially collected from the participants in one collection bag (120 mL) (sample-1, control). Within 2 min, the patients ingested an aqueous solution (200 mL) containing 75 mg of 13C-urea and 4.0 g of citric acid powder with added edulcorant. A second exhaled air collection was performed 15 min after ingestion of the substrate (sample-2). Each pair of collected material was analyzed by non-dispersive, isotope-selective infrared spectroscopythe (BreathID HP Lab System®, Exalenz Bioscience, Israel, now Meridian Bioscience, USA). Patients were considered positive for *H. pylori* when they had a delta over baseline (DOB) equal to or greater than 5‰^31^. All tests were carried out during the three days of SBAD, and the samples were sent for analysis to the Hospital ofClinics of UFMG, Belo Horizonte City.

### Statistics

Descriptive statistical techniques, including measures of central tendencies and variability, were used. Continuous variables are presented as arithmetic means with their respective standard deviations. Differences in proportions were assessed using Fisher’s exact test or the chi-square test, as appropriate. Qualitative variables are described as percentages and 95% confidence intervals. Statistical significance was set at *P*<0.05.

## RESULTS

**FIGURE 1:**
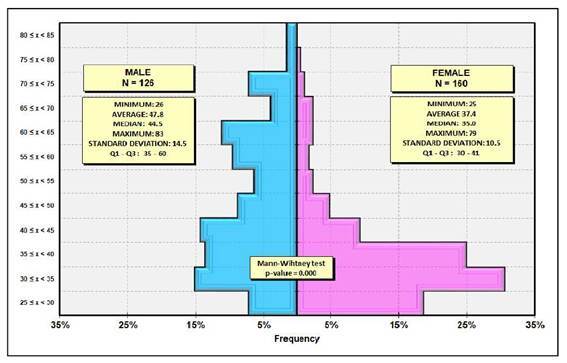
Distribution of the participants (N=286) by sex, age, and *P*-value.

A total of 299 participants were initially included in the study, with 13 excluded from the analysis: ten for not meeting the study inclusion criteria or incomplete questionnaire responses, and three due to sample automatically rejection by the equipment. Thus, 286 participants (160 females and 126 males a mean age of 42 years (min 25, max 83, SD 13) completed the study (Figure 1). Females were significantly more frequent and younger than males (*P*=0.000). Table 1 shows demographic and daily gastroenterological data.

**TABLE 1:** Demographic and daily-practice gastroenterological data of all participants (N=286).

Variable		n	%
**Etnicity**	White and mixed	273	95.5
	Asian	7	2.4
	Black	5	1.7
	Non-informed	1	0.3
**Region of birth in Brazil**	Southeastern	118	41.3
	Northeastern	76	26.6
	Southern	62	21.7
	Central Western	19	6.6
	Northern	9	3.1
**Body mass index (BMI)**	Underweight	2	0.7
	Normal	156	54.5
	Overweight	90	31.5
	Obsesity Class I	36	12.6
	Obesity Class II	1	0.3
	Obesity Class III	1	0.3
**Tabagism**	No	279	97.6
	Yes	7	2.4
**Medical activity**	Gastroenterology	151	52.8
	Gastroendoscopy	62	21.7
	Endoscopy	31	10.8
	Surgery/endoscopy	14	4.9
	Digestive surgery	13	4.5
	Gastro/surgery	6	2.1
	Surgery/endoscopy	2	0.7
	Other	7	2.4
**Time in speciality**	Up to 5 years (y)	127	44.4
	Between 5 to 10 y	49	17.1
	Between 10 to15 y	27	9.4
	Between 15 to 20 y	17	5.9
	More than 20 y	66	23.1

**FIGURE 2:**
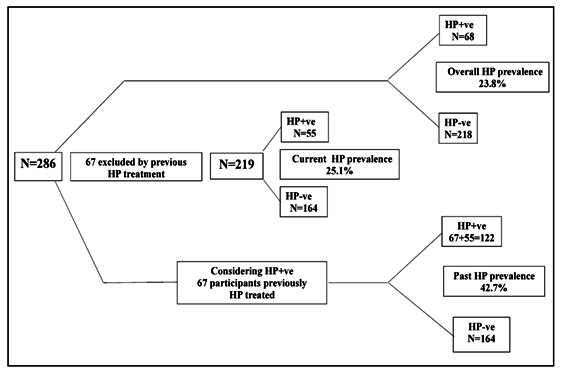
Overall, current and past HP prevalences observed in the study.

Figure 2 shows that 286 study participants, 218 tested negative for the UBT and 68 tested positive, with an overall prevalence of 23.8%. If we excluded all 67 participants who reported prior treatment for *H. pylori* infection (54 HP-ve and 13 HP+ve) from the analysis of our sample, our sample of 219 participants presented a current prevalence of 25.1% (55 HP+ve and 164 HP-ve). Considering a sample of 286 participants, the real prevalence was 42.7% when considering all 67 participants previously treated as HP+ve (122 HP+ positive and 164 HP-ve). To meet the aims of this study, the results presented below correspond to the analyses obtained from 219 participants who had never undergone previous treatment to eradicate *H. pylori*, representing the current prevalence in our sample.

Overall HP prevalence: All participants included (n=286). Current HP prevalence (n=219): only participants never submitted to HP treatment. Past prevalence (n=286): All participants included, considering HP+ve all those previously treated.

Table 2 shows the association between current *H. pylori* prevalence and the studied variables. Although *H. pylori* prevalence was significantly higher in males (34.7%) than females (17.7%) (*P*=0.004), sex may be a confounding variable because females are significantly younger than males (FIGURE 1, *P*=0.000). The joint analysis of *H. pylori* prevalence in overweight and obese participants was significantly higher than that observed in participants with normal and underweight BMI (32.3% and 19.3%, respectively; *P*=0.031). The joint analysis of HP prevalence in participants born in the northern and north eastern regions (34.5%), with lower human development index (HDI) of 0.667 and 0.663, respectively, was significantly higher than the prevalence observed in those born in the southern, south eastern, and central-western regions (20.8%), with higher HDI of 0.754, 0.766, and 0.757, respectively (*P*=0.037).

**TABLE 2:** Association between current HP prevalence and variables studied among the 219 participants.

Variables studied	n	%	HP prevalence (%)
Gender*			
Female	124	56.6	17.7
Male	95	43.4	34.7
Age range (years)**			
25-34	89	40.7	14.6
35-44	73	33.3	23.3
45-54	22	10.0	36.4
55-64	23	10.5	43.5
>64	12	5.5	58.3
**BMI [weight(kg)/height(cm)** ^2^ **]** ^#^			
Underweight + Normal	119	54.3	19.3
Overweight + Obesity	100	45.7	32.0
**Region of birth (Brasil)** ^# #^			
North+Northeast	58	26.7	34.5
Midwest+Southeast+South	159	73.3	20.8
**Endoscopy-related activities** ^&^			
No	138	38.1	23.2
Yes	81	61.9	28.4

BMI: body mass index. *χ^2^ (sig, *P*-value: 0.004); **χ^2^ (sig, *P*-value: 0.001; ^#^χ^2^ (*P*-value: sig, 0.031); ^##^χ^2^ (*P*-value: sig, 0.037); ^&^χ^2^ (non-sig, *P*-value: 0. 391).

**FIGURE 3:**
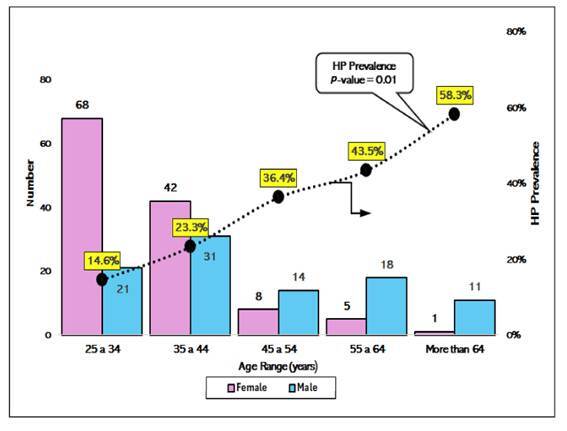
Number of participants and HP prevalence by age (N=219).

Figure 3 illustrates a significant increase in the prevalence of *H. pylori* infection with age, reaching 58.3% in participants aged >64 years (*P*=0.01). 

**FIGURE 4:**
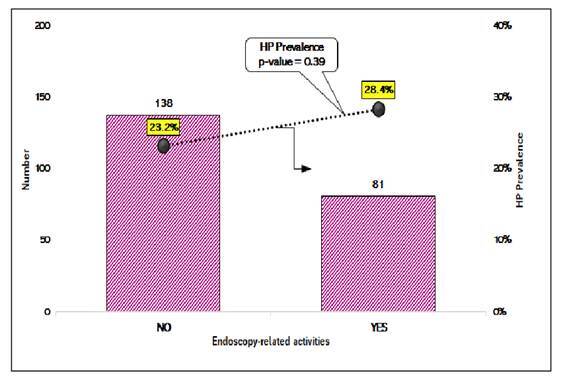
Number of participants and HP prevalence by endoscopy-related activities (N=219).

Figure 4 shows the overall prevalence of *H. pylori* among participants who did or did not perform endoscopic procedures in their daily activities (28.4% and 23.2%, respectively), with no statistically significant difference (*P*=0.39).

**FIGURE 5:**
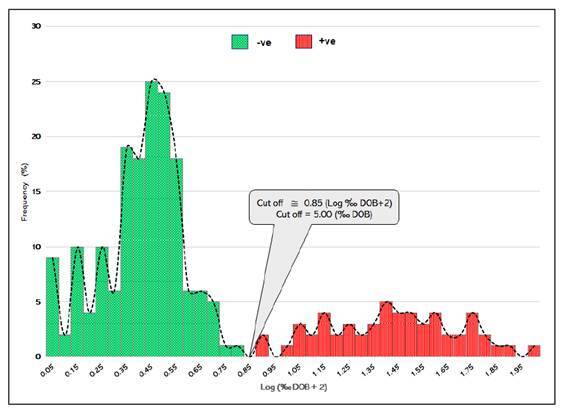
Urea breath test values in HP+ve and HP-ve participants (N=219).

DOB: delta over baseline; Log: logaritm.

Figure 5 shows the performance of the UBT in discriminating between *H. pylori*-positive and *H. pylori*-negative patients. The results expressed on a logarithmic scale plus a constant 2 to overcome the possibility of the test results presenting negative DOB values showed an evident separation between the infected and non-infected individuals.

## DISCUSSION

Our study confirmed a downward trend in the prevalence of *H. pylori* infection in adults worldwide over the past decade^32^. The current prevalence observed in adults (21.5%) is lower than that observed in previous decades in Brazil, at approximately 50-70% of the general adult population^33^ ^-^ ^35^. Recent studies in adults and children in Brazil have also shown a prevalence *of H. pylori* infection of approximately 20-50%^22^ ^,^ ^36^. Although decreasing, the finding of one in four Brazilian gastroenterologists infected with *H. pylori* remains relevant.

The reduction in the prevalence of *H. pylori* infection has been attributed to an improvement in the socioeconomic conditions of the population, including hygiene and housing standards, with the treatment of infected individuals also considered a contributing factor. We found significant differences in the *H. pylori* prevalence among gastroenterologists in Brazil. The joint analysis of *H. pylori* prevalence in participants born in the northern and north eastern regions (34.5%), with lower human development index (HDI) of 0.667 and 0.663^37^, respectively, was significantly higher than the prevalence observed in those born in the southern, south eastern, and central-western regions (20.8%), with higher HDI of 0.754, 0.766, and 0.757, respectively (*P*=0.037). The significantly higher prevalence observed in older individuals than in younger participants is explained by most (90%) *H. pylori* infections being acquired in childhood and persisting throughout life rather than by a higher risk of infection at an older age^38^. Our findings show that overweight and obese participants have a higher prevalence of infection than normal weight or underweight participants, as confirmed by a recent systematic review and case-control meta-analysis, although the potential mechanisms involved in this association remain controversial^39^.

Our findings showed no significant difference in the rate of *H. pylori* infection between gastroenterologists who did and did not perform endoscopic procedures. Although the occupational risk of acquiring *H. pylori* infection is controversial, a systematic review of 98 studies suggested that healthcare professionals, especially those linked to the gastrointestinal area, are exposed to greater risk, suggesting a possible iatrogenic route of transmission^13^. The detection of *H. pylori* DNA in the vomitus, saliva, dental plaque, gastric juice, and feces favors its transmission via endoscopic procedures^8^ ^-^ ^11^. However, initial studies evaluating whether gastroendoscopists are at an increased risk of *H. pylori* infection were contradictory^40^ ^,^ ^41^. The explanation for our findings and those confirmed by other studies^41^ ^,^ ^42^ that did not find dif­ferences in prevalence between gastroendoscopists and gastroenterologists is still incomplete and may involve a small volume of infectious inocula sprayed onto the mucous membranes of the mouth of the endoscopist, and certainly, due to strict adherence to the disinfection and protection protocols with gloves and masks currently adopted worldwide^43^ ^,^ ^44^.

Our study has limitations and strengths. The study population consisted of Brazilian gastroenterologists and gastroendoscopists present at the SBAD-2022 and members of Brazilian societies of gastroenterology and/or digestive endoscopy and/or digestive surgery. Although other professionals may work in these specialties without being members of these societies, they are mainly responsible for certifying specialists in Brazil and are therefore representative of the populations studied. The absence of a control group from the general population, preferably not linked to medical care, could highlight the significant differences in the prevalence of *H. pylori* in Brazil, and new studies may be needed in this area. To the best of our knowledge, this is the first study to explore *H. pylori* infection as an occupational risk factor among gastroenterologists and gastroendoscopists in Brazil. Moreover, it is worth highlighting the use of UBT as a noninvasive diagnostic method. Its high accuracy, with sensitivity and specificity in the order 90-95%^6^ ^,^ ^14^ ^,^ ^15^, surpasses the serological methods used in most previous studies and has limitations in defining cut-off values and sensitivity depending on variations in reagents and laboratory conditions^12^. Combined with an adequate sample size for the study, its use makes the obtained results robust.

In conclusion, the prevalence of *H. pylori* infection among Brazilian gastroenterologists is moderate, with one in four professionals still infected with the bacteria. The prevalence of *H. pylori* infection increa­ses with age, and is higher in overweight and obese individuals. Performing endoscopic procedures does not appear to increase the risk of acquiring infections among gastroenterologists in Brazil.
